# Crystal structure of bis­{2-[(*E*)-(4-fluoro­benz­yl)imino­meth­yl]phenolato-κ^2^ *N*,*O*}nickel(II)

**DOI:** 10.1107/S1600536814020546

**Published:** 2014-09-24

**Authors:** Amalina Mohd Tajuddin, Hadariah Bahron, Rohazila Mohammad Hanafiah, Nazlina Ibrahim, Hoong-Kun Fun, Suchada Chantrapromma

**Affiliations:** a Faculty of Applied Sciences, Universiti Teknologi MARA, 40450 Shah Alam, Selangor, Malaysia; b DDH CoRe, Universiti Teknologi MARA, 40450 Shah Alam, Selangor, Malaysia; c School of Biosciences and Biotechnology, Faculty of Science and Technology, Universiti Kebangsaan, 43600 Bangi, Selangor, Malaysia; d X-ray Crystallography Unit, School of Physics, Universiti Sains Malaysia, 11800 USM, Penang, Malaysia; e Department of Pharmaceutical Chemistry, College of Pharmacy, King Saud University, PO Box 2457, Riyadh 11451, Saudi Arabia; f Department of Chemistry, Faculty of Science, Prince of Songkla University, Hat-Yai, Songkhla 90112, Thailand

**Keywords:** Crystal structure, Ni(II) complex, NO donors, Schiff base, anti­bacterial activity

## Abstract

The asymmetric unit of the title complex, [Ni(C_14_H_11_FNO)_2_], contains one-half of the mol­ecule with the Ni^II^ cation lying on an inversion centre coordinated by a bidentate Schiff base anion. The cationic Ni^II^ center is in a distorted square-planar coordination environment chelated by the imine N and phenolate O donor atoms of the two Schiff base ligands. The N and O donor atoms of the two ligands are mutually *trans* with Ni—N and Ni—O bond lengths of 1.9242 (10) and 1.8336 (9) Å, respectively. The fluoro­phenyl ring is almost orthogonal to the coordination plane and makes a dihedral angle of 82.98 (7)° with the phenolate ring. In the crystal, mol­ecules are linked into screw chains by weak C—H⋯F hydrogen bonds. Additional C—H⋯π contacts arrange the mol­ecules into sheets parallel to the *ac* plane.

## Abstract 2

In the square-planar [Ni(C_14_H_11_FNO)_2_] complex, weak C—H⋯F and C—H⋯π inter­actions play an important role in the mol­ecular self-assembly, resulting in the formation of 2D mol­ecular sheets which are stacked along the *b* axis.

## Chemical context   

Schiff base ligands are well-known and important compounds because of their wide range of biological activities and uses in industrial systems (Feng *et al.*, 2013 ▶; Kumar *et al.*, 2010 ▶; Liu *et al.*, 2005 ▶) as well as being versatile ligands for transition metals. Transition metal complexes with Schiff base ligands, especially those of Pd^II^ and Ni^II^, have been shown to display a variety of structural features and, in some cases, exhibit inter­esting reactivity. In particular they can be photoluminescent (Guo *et al.*, 2013*a* ▶) and are used as catalysts for many organic reactions such as Heck and Suzuki cross-coupling reactions (Kumari *et al.*, 2012 ▶; Teo *et al.*, 2011 ▶).
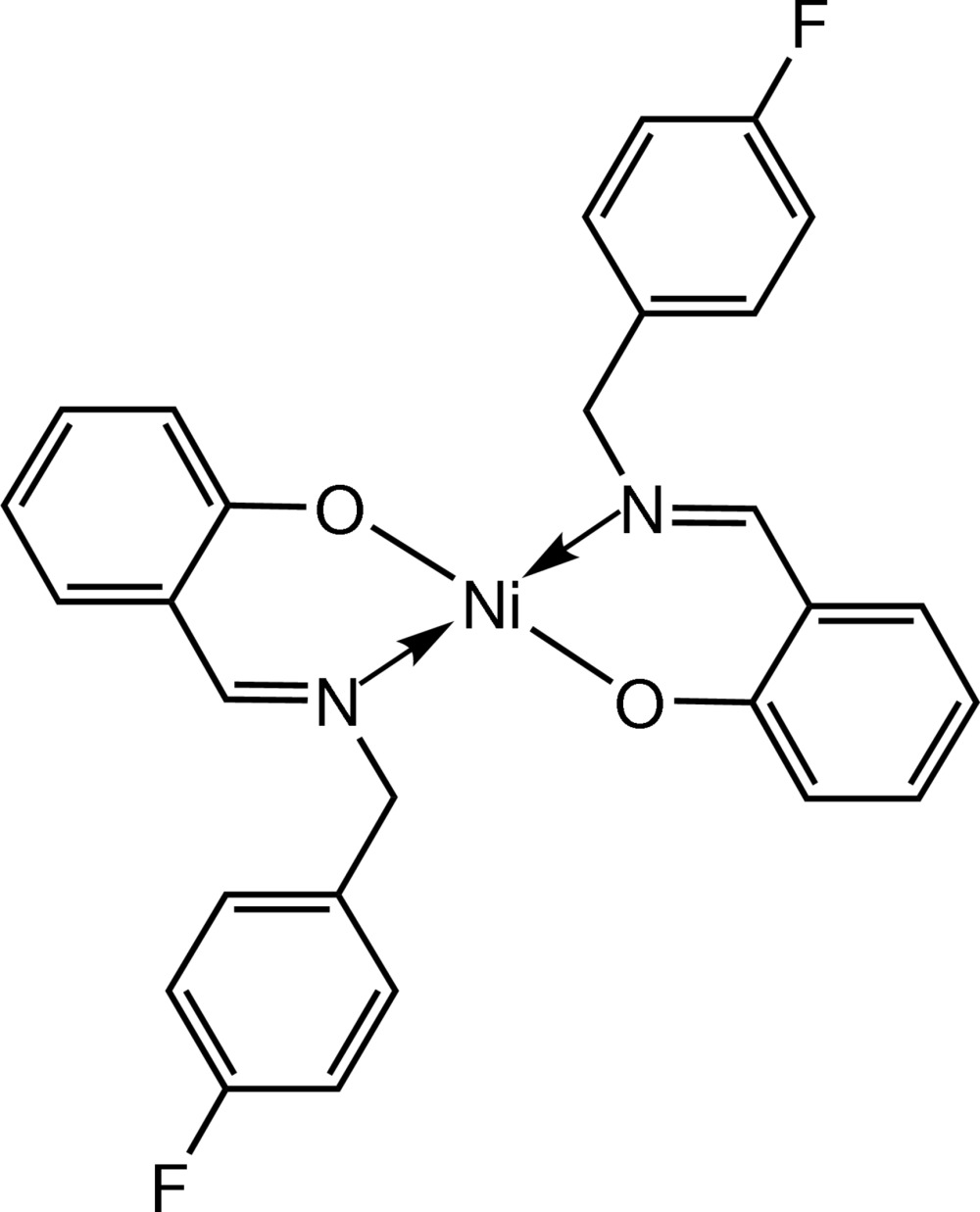


**Figure 1:**
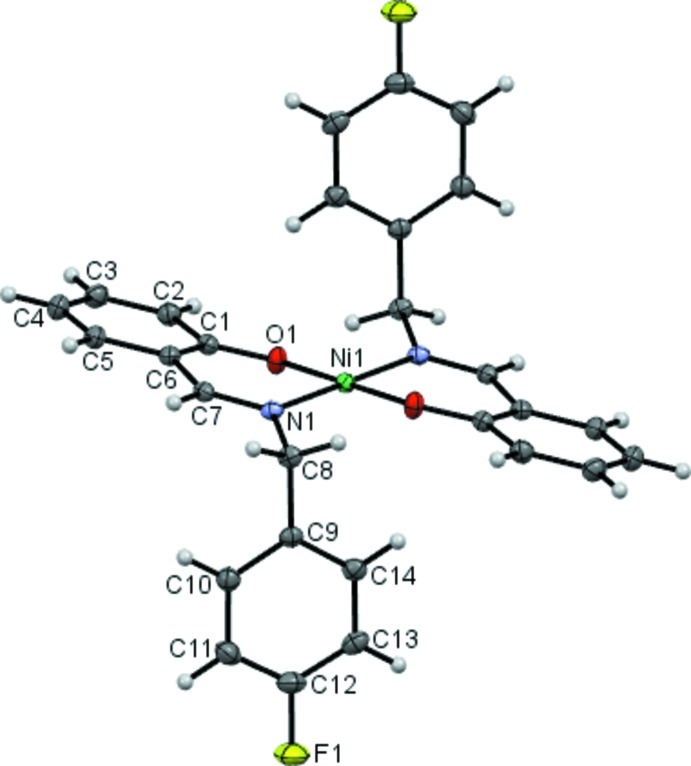
The mol­ecular structure of (1), showing 50% probability displacement ellipsoids and the atom-numbering scheme. The labelled atoms are related to the unlabelled atoms of the Schiff base ligands by the symmetry code: 1 − *x*, −*y*, 1 − *z*.

 In our previous studies, we reported the syntheses and crystal structures of two related Schiff base complexes, bis­{2-[(*E*)-(4-fluoro­benz­yl)imino­meth­yl]-6-meth­oxy­phenolato-κ^2^ *N,O* ^1^}nickel(II) (Bahron *et al.*, 2011 ▶) and bis­{2-[(*E*)-(4-meth­oxy­benz­yl)imino­meth­yl]phenolato-κ^2^ *N,O* ^1^}nickel(II) (Bahron *et al.*, 2014 ▶). In this article, we report the successful synthesis of another Schiff base–Ni^II^ complex, [Ni(C_14_H_11_FNO)_2_] (1), and its characterization by spectros­copy and elemental analysis. Crystal structure determination confirms the binding mode of the [(4-fluoro­benz­yl)imino­meth­yl]phenolate ligand to the Ni^II^ cation (Fig. 1 ▶). The title complex was also tested for anti­bacterial activity, and found to be only weakly active.

## Structural commentary   

The asymmetric unit of (1) contains one-half of the mol­ecule with the Ni^II^ cation lying on an inversion centre and the Schiff base anion acting as an *N*,*O-*bidentate chelate ligand (Fig. 1 ▶). The cation binds to the N and the O atoms of two symmetry-related Schiff base ligand such that the N and O atoms are mutually *trans*. The N_2_O_2_ donor sets of the two chelating Schiff base ligands in the equatorial plane around Ni1 adopt a slightly distorted square planar coordination geometry with the angles O1—Ni1—N1 = 92.56 (4)° and O1—Ni1—N1^i^ = 87.44 (4)° [symmetry code: (i) 1 − *x*, −*y*, 1 − *z*]. As expected under inversion symmetry, the *trans* angles (N11—Ni1—N1^i^ and O1—Ni1—O1^i^) are found to be linear. The Ni1—N1 and Ni1—O1 distances in the N_2_O_2_ coordination plane are 1.9242 (10) Å and 1.8336 (9) Å, respectively. These compare well with those observed in the two other closely related Ni^II^ complexes with N_2_O_2_ coordinating Schiff base ligands (Bahron *et al.*, 2011 ▶; 2014 ▶). The Ni1/O1/C1/C6/C7/N1 ring adopts an envelope conformation with the Ni1 atom displaced by 0.3885 (5) Å from the O1/C1/C6/C7/N1 plane, with the puckering parameters *Q* = 0.2429 (10) Å, θ = 65.3 (3) and ϕ = 4.0 (3)°. Other bond lengths and angles observed in the structure are also normal. The fluoro­phenyl ring (C9–C14) makes a dihedral angle of 82.98 (7)° with the phenolate ring (C1–C6).

## Supra­molecular features   

**Figure 2:**
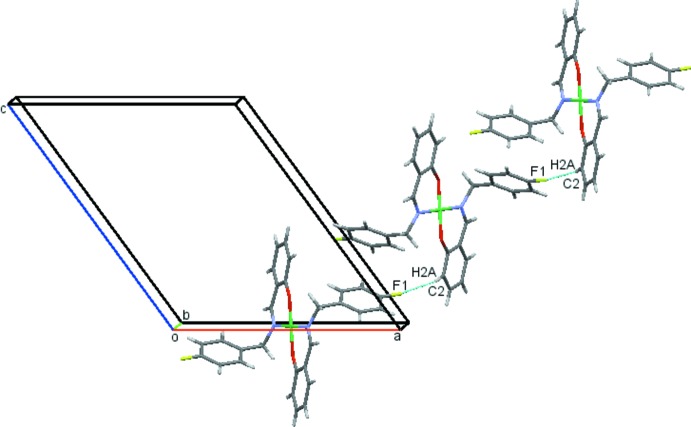
Screw chains of mol­ecules of (1) linked by C—H⋯F contacts drawn as dashed lines.

**Figure 3:**
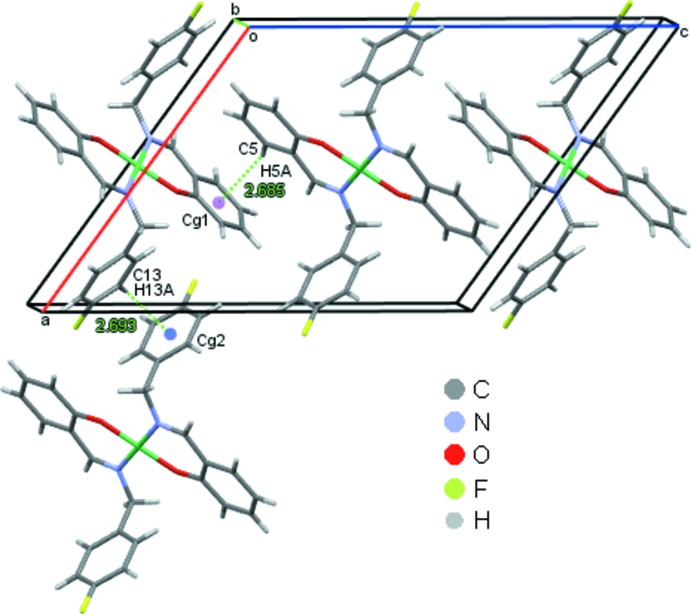
C—H⋯π contacts for (1) drawn as dotted lines with ring centroids shown as coloured spheres. *Cg*1 and *Cg*2 are the centroids of the C1–C6 and C9–C14 rings, respectively.

**Figure 4:**
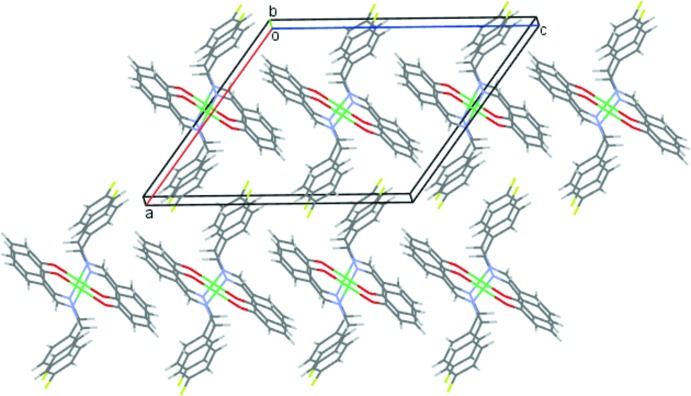
The packing of (1) viewed along the *b* axis showing mol­ecular sheets of the Ni^II^ complex.

In the crystal packing, the mol­ecules are linked into screw chains by weak C2—H2*A*⋯F1 inter­actions (Fig. 2 ▶, Table 1 ▶). C—H⋯π inter­actions involving both the fluoro­phenyl and the phenolate rings, C5—H5*A*⋯*Cg*1 and C13—H13*A*⋯*Cg*2, connect the mol­ecules into chains along the *c-*axis direction (Fig. 3 ▶, Table 1 ▶). They also contribute to the formation of sheets parallel to the *ac* plane, which are further stacked along the *b* axis as shown in Fig. 4 ▶.

## Database survey   

A search of the Cambridge Database (Version 5.35, November 2013 with 3 updates) revealed a total of 1191 Ni^II^ complexes with an NiN_2_O_2_ coordination sphere. No fewer than 333 of these had the Ni atom chelated by two 3-(imino­meth­yl)phenolate residues. No corresponding structures with a benzyl or substituted benzyl unit bound to the imino N atom were found. However extending the search to allow additional substitution on the phenolate ring resulted in eight discrete structures including the two closely related structures mentioned previously (Bahron *et al.*, 2011 ▶, 2014 ▶), and several other related complexes (see, for example Guo *et al.* 2013*a* ▶,*b* ▶; Senol *et al.* 2011 ▶; Chen *et al.* 2010 ▶).

## Synthesis and crystallization   

An ethano­lic solution of 4-fluoro­benzyl­amine (4 mmol, 0.5010 g) was added to salicyl­aldehyde (4 mmol, 0.4970 g), dissolved in absolute ethanol (2 ml), forming a bright-yellow solution. The mixture was heated under reflux for an hour to produce the ligand, (*E*)-2-[(4-fluoro­benzyl­imino)­meth­yl]phenol. Nickel(II) acetate tetra­hydrate (2 mmol, 0.4983 g) was dissolved separately in absolute ethanol (10 ml) and added to a flask containing the cooled ligand solution. The mixture was stirred and refluxed for 3 h upon which a dark-green solid formed. This was filtered off, washed with ice-cold ethanol and air-dried at room temperature. The solid product was recrystallized from chloro­form, yielding green crystals. Yield 68.6%; m.p. 471–473 K. Analytical data for C_28_H_22_F_2_N_2_O_2_Ni: C, 65.28; H, 4.30; N, 5.44. Found: C, 65.87; H, 4.39; N, 5.55. IR (KBr, cm^−1^): ν(C=N) 1612 (*s*), ν(C—N) 1390 (*w*), ν(C—O) 1221 (*s*), ν(Ni—N) 597 (*w*), ν(Ni—O) 451 (*w*). The infrared spectra of the title complex revealed a strong band of 1612 cm^−1^ in the spectrum assignable to C=N stretching frequency upon complexation (Nair *et al.*, 2012 ▶). The appearance of new bands at 451 and 597 cm^−1^ in the spectrum of the title complex attributable to Ni—O and Ni—N vibrations, respectively, supports the suggestion above of the participation of the N atom of the imine group and O atom of the phenolic group of the ligand in the complexation with Ni^II^ cation (Ouf *et al.*, 2010 ▶). Accordingly, it can be deduced that the ligand binds to the Ni^II^ cation in an *N*,*O-*bidentate fashion in 2:1 ratio.

An anti­bacterial activity investigation of the title complex against *B. subtilis*, *S. aureus* and *E. coli* showed very mild or no inhibition with clear inhibition diameters of 7–8 mm at the highest concentration of 50 μ*M*. The negative control of a 9:1 mixture of DMSO:acetone and the positive control of 30 U of chloramphenicol showed inhibition diameters of 6 mm and 20 mm, respectively.

## Refinement   

Crystal data, data collection and structure refinement details are summarized in Table 2 ▶. All H atoms were positioned geometrically and allowed to ride on their parent atoms, with *d*(C—H) = 0.95 Å for aromatic and 0.99 Å for CH_2_ hydrogen atoms. The *U*_iso_ values were constrained to be 1.2*U*_eq_ of the carrier atoms.

## Supplementary Material

Crystal structure: contains datablock(s) global, I. DOI: 10.1107/S1600536814020546/sj5425sup1.cif

Structure factors: contains datablock(s) I. DOI: 10.1107/S1600536814020546/sj5425Isup2.hkl

CCDC reference: 1024161

Additional supporting information:  crystallographic information; 3D view; checkCIF report

## Figures and Tables

**Table 1:** Hydrogen-bond geometry (Å, °) *Cg*1 and *Cg*2 are the centroids of the C1–C6 and C9–C14 rings, respectively.

*D*—H⋯*A*	*D*—H	H⋯*A*	*D*⋯*A*	*D*—H⋯*A*
C8—H8*A*⋯O1^i^	0.99	2.19	2.7300 (18)	113
C14—H14*A*⋯O1^i^	0.95	2.52	3.212 (2)	130
C2—H2*A*⋯F1^ii^	0.95	2.65	3.5312 (19)	155
C5—H5*A*⋯*Cg*1^iii^	0.95	2.69	3.4010 (18)	133
C13—H13*A*⋯*Cg*2^iv^	0.95	2.69	3.4252 (13)	134

Symmetry codes: (i) 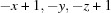; (ii) 
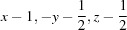
; (iii) 
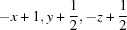
; (iv) 
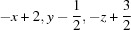
.

**Table 2:** Experimental details

Crystal data	
Chemical formula	[Ni(C_14_H_11_FNO)_2_]
*M*_r_	515.17
Crystal system, space group	Monoclinic, *P*2_1_/*c*
Temperature (K)	100
*a*, *b*, *c* (Å)	13.8611 (3), 5.83340 (1), 16.9942 (3)
β (°)	125.998 (1)
*V* (Å^3^)	1111.70 (4)
*Z*	2
Radiation type	Mo *K*α
μ (mm^−1^)	0.92
Crystal size (mm)	0.47 × 0.19 × 0.11

Data collection	
Diffractometer	Bruker APEXII CCD area detector
Absorption correction	Multi-scan (*SADABS*; Bruker, 2009 ▶)
*T*_min_, *T*_max_	0.674, 0.906
No. of measured, independent and observed [*I* > 2σ(*I*)] reflections	13419, 3235, 2896
*R*_int_	0.024
(sin θ/λ)_max_ (Å^−1^)	0.703

Refinement	
*R*[*F* ^2^ > 2σ(*F* ^2^)], *wR*(*F* ^2^), *S*	0.028, 0.072, 1.05
No. of reflections	3235
No. of parameters	160
H-atom treatment	H-atom parameters constrained
Δρ_max_, Δρ_min_ (e Å^−3^)	0.45, −0.49

Computer programs: *APEX2* and *SAINT* (Bruker, 2009 ▶), *SHELXTL* (Sheldrick, 2008 ▶), *PLATON* (Spek, 2009 ▶), *Mercury* (Macrae *et al.*, 2006 ▶) and *publCIF* (Westrip, 2010 ▶).

## supplementary crystallographic information

### Crystal data

**Table:**

[Ni(C_14_H_11_FNO)_2_]	*F*(000) = 532
*M**_r_* = 515.17	*D*_x_ = 1.539 Mg m^−^^3^
Monoclinic, *P*2_1_/*c*	Melting point = 471–476 K
Hall symbol: -P 2ybc	Mo *K*α radiation, λ = 0.71073 Å
*a* = 13.8611 (3) Å	Cell parameters from 3235 reflections
*b* = 5.83340 (1) Å	θ = 1.8–30.0°
*c* = 16.9942 (3) Å	µ = 0.92 mm^−^^1^
β = 125.998 (1)°	*T* = 100 K
*V* = 1111.70 (4) Å^3^	Plate, green
*Z* = 2	0.47 × 0.19 × 0.11 mm

### Data collection

**Table:**

Bruker APEXII CCD area detector diffractometer	3235 independent reflections
Radiation source: sealed tube	2896 reflections with *I* > 2σ(*I*)
Graphite monochromator	*R*_int_ = 0.024
φ and ω scans	θ_max_ = 30.0°, θ_min_ = 1.8°
Absorption correction: multi-scan (*SADABS*; Bruker, 2009)	*h* = −19→19
*T*_min_ = 0.674, *T*_max_ = 0.906	*k* = −8→8
13419 measured reflections	*l* = −23→23

### Refinement

**Table:**

Refinement on *F*^2^	Primary atom site location: structure-invariant direct methods
Least-squares matrix: full	Secondary atom site location: difference Fourier map
*R*[*F*^2^ > 2σ(*F*^2^)] = 0.028	Hydrogen site location: inferred from neighbouring sites
*wR*(*F*^2^) = 0.072	H-atom parameters constrained
*S* = 1.05	*w* = 1/[σ^2^(*F*_o_^2^) + (0.0322*P*)^2^ + 0.7123*P*] where *P* = (*F*_o_^2^ + 2*F*_c_^2^)/3
3235 reflections	(Δ/σ)_max_ = 0.001
160 parameters	Δρ_max_ = 0.45 e Å^−^^3^
0 restraints	Δρ_min_ = −0.49 e Å^−^^3^

### Special details

**Table:**

Experimental. The crystal was placed in the cold stream of an Oxford Cryosystems Cobra open-flow nitrogen cryostat (Cosier & Glazer, 1986) operating at 100.0 (1) K.
Geometry. All esds (except the esd in the dihedral angle between two l.s. planes) are estimated using the full covariance matrix. The cell esds are taken into account individually in the estimation of esds in distances, angles and torsion angles; correlations between esds in cell parameters are only used when they are defined by crystal symmetry. An approximate (isotropic) treatment of cell esds is used for estimating esds involving l.s. planes.
Refinement. Refinement of F^2^ against ALL reflections. The weighted R-factor wR and goodness of fit S are based on F^2^, conventional R-factors R are based on F, with F set to zero for negative F^2^. The threshold expression of F^2^ > 2sigma(F^2^) is used only for calculating R-factors(gt) etc. and is not relevant to the choice of reflections for refinement. R-factors based on F^2^ are statistically about twice as large as those based on F, and R- factors based on ALL data will be even larger.

### Fractional atomic coordinates and isotropic or equivalent isotropic displacement parameters (Å^2^)

**Table:**

	*x*	*y*	*z*	*U*_iso_*/*U*_eq_	
Ni1	0.5000	0.0000	0.5000	0.01062 (7)	
F1	1.07321 (8)	−0.19874 (17)	0.63754 (7)	0.0297 (2)	
N1	0.58764 (8)	0.26207 (18)	0.50481 (7)	0.01200 (19)	
O1	0.41017 (8)	−0.01507 (15)	0.36693 (7)	0.01520 (18)	
C1	0.38843 (10)	0.1456 (2)	0.30461 (9)	0.0133 (2)	
C2	0.29659 (11)	0.1091 (2)	0.20455 (9)	0.0160 (2)	
H2A	0.2528	−0.0303	0.1840	0.019*	
C3	0.27066 (11)	0.2756 (2)	0.13703 (9)	0.0174 (2)	
H3A	0.2085	0.2490	0.0704	0.021*	
C4	0.33373 (12)	0.4832 (2)	0.16425 (10)	0.0175 (2)	
H4A	0.3147	0.5959	0.1168	0.021*	
C5	0.42385 (11)	0.5206 (2)	0.26118 (9)	0.0151 (2)	
H5A	0.4676	0.6600	0.2804	0.018*	
C6	0.45190 (10)	0.3546 (2)	0.33205 (8)	0.0125 (2)	
C7	0.55207 (10)	0.3936 (2)	0.43090 (9)	0.0125 (2)	
H7A	0.5968	0.5294	0.4434	0.015*	
C8	0.70465 (10)	0.3283 (2)	0.59589 (9)	0.0134 (2)	
H8A	0.7024	0.3042	0.6524	0.016*	
H8B	0.7198	0.4930	0.5933	0.016*	
C9	0.80423 (10)	0.1869 (2)	0.60823 (8)	0.0133 (2)	
C10	0.86635 (11)	0.2669 (2)	0.57233 (9)	0.0166 (2)	
H10A	0.8456	0.4110	0.5402	0.020*	
C11	0.95823 (11)	0.1396 (3)	0.58258 (10)	0.0202 (3)	
H11A	1.0010	0.1957	0.5588	0.024*	
C12	0.98511 (11)	−0.0701 (3)	0.62825 (10)	0.0196 (3)	
C13	0.92625 (11)	−0.1569 (2)	0.66508 (9)	0.0174 (2)	
H13A	0.9471	−0.3019	0.6965	0.021*	
C14	0.83553 (11)	−0.0255 (2)	0.65471 (9)	0.0152 (2)	
H14A	0.7942	−0.0816	0.6798	0.018*	

### Atomic displacement parameters (Å^2^)

**Table:**

	*U*^11^	*U*^22^	*U*^33^	*U*^12^	*U*^13^	*U*^23^
Ni1	0.01005 (10)	0.01029 (11)	0.01063 (11)	−0.00089 (7)	0.00557 (8)	0.00019 (7)
F1	0.0240 (4)	0.0360 (5)	0.0340 (5)	0.0125 (4)	0.0197 (4)	0.0035 (4)
N1	0.0105 (4)	0.0115 (5)	0.0129 (4)	−0.0003 (3)	0.0063 (4)	−0.0010 (4)
O1	0.0168 (4)	0.0142 (4)	0.0122 (4)	−0.0036 (3)	0.0072 (3)	0.0005 (3)
C1	0.0123 (5)	0.0149 (5)	0.0138 (5)	0.0010 (4)	0.0084 (4)	0.0009 (4)
C2	0.0141 (5)	0.0177 (6)	0.0148 (5)	−0.0015 (4)	0.0077 (5)	−0.0003 (4)
C3	0.0141 (5)	0.0218 (6)	0.0136 (5)	0.0016 (4)	0.0066 (4)	0.0010 (5)
C4	0.0176 (6)	0.0188 (6)	0.0157 (6)	0.0029 (4)	0.0096 (5)	0.0049 (5)
C5	0.0153 (5)	0.0144 (6)	0.0167 (6)	0.0012 (4)	0.0100 (5)	0.0022 (4)
C6	0.0115 (5)	0.0133 (5)	0.0135 (5)	0.0014 (4)	0.0077 (4)	0.0010 (4)
C7	0.0122 (5)	0.0115 (5)	0.0158 (5)	−0.0002 (4)	0.0093 (4)	−0.0005 (4)
C8	0.0119 (5)	0.0116 (5)	0.0140 (5)	−0.0017 (4)	0.0062 (4)	−0.0020 (4)
C9	0.0103 (5)	0.0149 (5)	0.0114 (5)	−0.0012 (4)	0.0045 (4)	−0.0015 (4)
C10	0.0154 (5)	0.0179 (6)	0.0156 (5)	−0.0010 (4)	0.0086 (5)	0.0007 (4)
C11	0.0174 (6)	0.0271 (7)	0.0192 (6)	−0.0004 (5)	0.0126 (5)	−0.0003 (5)
C12	0.0139 (5)	0.0249 (7)	0.0182 (6)	0.0039 (5)	0.0085 (5)	−0.0020 (5)
C13	0.0139 (5)	0.0162 (6)	0.0161 (6)	0.0013 (4)	0.0054 (5)	−0.0002 (4)
C14	0.0123 (5)	0.0152 (6)	0.0155 (5)	−0.0018 (4)	0.0068 (4)	−0.0008 (4)

### Geometric parameters (Å, º)

**Table:**

Ni1—O1^i^	1.8336 (9)	C5—H5A	0.9500
Ni1—O1	1.8336 (9)	C6—C7	1.4351 (16)
Ni1—N1^i^	1.9242 (10)	C7—H7A	0.9500
Ni1—N1	1.9242 (10)	C8—C9	1.5133 (16)
F1—C12	1.3613 (15)	C8—H8A	0.9900
N1—C7	1.2967 (16)	C8—H8B	0.9900
N1—C8	1.4915 (15)	C9—C14	1.3943 (17)
O1—C1	1.3097 (15)	C9—C10	1.3960 (17)
C1—C6	1.4130 (17)	C10—C11	1.3937 (18)
C1—C2	1.4187 (17)	C10—H10A	0.9500
C2—C3	1.3801 (18)	C11—C12	1.378 (2)
C2—H2A	0.9500	C11—H11A	0.9500
C3—C4	1.4031 (19)	C12—C13	1.3834 (19)
C3—H3A	0.9500	C13—C14	1.3926 (17)
C4—C5	1.3794 (18)	C13—H13A	0.9500
C4—H4A	0.9500	C14—H14A	0.9500
C5—C6	1.4100 (17)		
			
O1^i^—Ni1—O1	180.0	N1—C7—C6	126.56 (11)
O1^i^—Ni1—N1^i^	92.56 (4)	N1—C7—H7A	116.7
O1—Ni1—N1^i^	87.44 (4)	C6—C7—H7A	116.7
O1^i^—Ni1—N1	87.44 (4)	N1—C8—C9	110.45 (9)
O1—Ni1—N1	92.56 (4)	N1—C8—H8A	109.6
N1^i^—Ni1—N1	180.00 (6)	C9—C8—H8A	109.6
C7—N1—C8	114.48 (10)	N1—C8—H8B	109.6
C7—N1—Ni1	123.90 (8)	C9—C8—H8B	109.6
C8—N1—Ni1	121.62 (8)	H8A—C8—H8B	108.1
C1—O1—Ni1	129.03 (8)	C14—C9—C10	118.57 (11)
O1—C1—C6	123.23 (11)	C14—C9—C8	121.18 (11)
O1—C1—C2	118.67 (11)	C10—C9—C8	120.25 (11)
C6—C1—C2	118.10 (11)	C11—C10—C9	121.36 (12)
C3—C2—C1	120.18 (12)	C11—C10—H10A	119.3
C3—C2—H2A	119.9	C9—C10—H10A	119.3
C1—C2—H2A	119.9	C12—C11—C10	117.89 (12)
C2—C3—C4	121.73 (12)	C12—C11—H11A	121.1
C2—C3—H3A	119.1	C10—C11—H11A	121.1
C4—C3—H3A	119.1	F1—C12—C11	118.81 (12)
C5—C4—C3	118.79 (12)	F1—C12—C13	118.24 (13)
C5—C4—H4A	120.6	C11—C12—C13	122.95 (12)
C3—C4—H4A	120.6	C12—C13—C14	118.04 (12)
C4—C5—C6	120.87 (12)	C12—C13—H13A	121.0
C4—C5—H5A	119.6	C14—C13—H13A	121.0
C6—C5—H5A	119.6	C13—C14—C9	121.18 (12)
C5—C6—C1	120.33 (11)	C13—C14—H14A	119.4
C5—C6—C7	118.85 (11)	C9—C14—H14A	119.4
C1—C6—C7	120.62 (11)		
			
O1^i^—Ni1—N1—C7	−161.69 (10)	C8—N1—C7—C6	171.36 (11)
O1—Ni1—N1—C7	18.31 (10)	Ni1—N1—C7—C6	−8.09 (17)
O1^i^—Ni1—N1—C8	18.90 (9)	C5—C6—C7—N1	177.97 (11)
O1—Ni1—N1—C8	−161.10 (9)	C1—C6—C7—N1	−7.20 (18)
N1^i^—Ni1—O1—C1	158.63 (10)	C7—N1—C8—C9	−97.31 (12)
N1—Ni1—O1—C1	−21.37 (10)	Ni1—N1—C8—C9	82.15 (11)
Ni1—O1—C1—C6	12.98 (17)	N1—C8—C9—C14	−87.37 (13)
Ni1—O1—C1—C2	−166.89 (9)	N1—C8—C9—C10	92.19 (13)
O1—C1—C2—C3	179.45 (11)	C14—C9—C10—C11	−0.29 (18)
C6—C1—C2—C3	−0.42 (17)	C8—C9—C10—C11	−179.86 (12)
C1—C2—C3—C4	0.48 (19)	C9—C10—C11—C12	0.9 (2)
C2—C3—C4—C5	−0.03 (19)	C10—C11—C12—F1	178.90 (12)
C3—C4—C5—C6	−0.47 (19)	C10—C11—C12—C13	−0.9 (2)
C4—C5—C6—C1	0.52 (18)	F1—C12—C13—C14	−179.49 (11)
C4—C5—C6—C7	175.37 (11)	C11—C12—C13—C14	0.3 (2)
O1—C1—C6—C5	−179.94 (11)	C12—C13—C14—C9	0.31 (19)
C2—C1—C6—C5	−0.07 (17)	C10—C9—C14—C13	−0.32 (18)
O1—C1—C6—C7	5.30 (18)	C8—C9—C14—C13	179.25 (11)
C2—C1—C6—C7	−174.83 (11)		

Symmetry code: (i) −*x*+1, −*y*, −*z*+1.

### Hydrogen-bond geometry (Å, º)

*Cg*1 and *Cg*2 are the centroids of the C1–C6 and C9–C14 rings, respectively.

**Table:**

*D*—H···*A*	*D*—H	H···*A*	*D*···*A*	*D*—H···*A*
C8—H8*A*···O1^i^	0.99	2.19	2.7300 (18)	113
C14—H14*A*···O1^i^	0.95	2.52	3.212 (2)	130
C2—H2*A*···F1^ii^	0.95	2.65	3.5312 (19)	155
C5—H5*A*···*Cg*1^iii^	0.95	2.69	3.4010 (18)	133
C13—H13*A*···*Cg*2^iv^	0.95	2.69	3.4252 (13)	134

Symmetry codes: (i) −*x*+1, −*y*, −*z*+1; (ii) *x*−1, −*y*−1/2, *z*−1/2; (iii) −*x*+1, *y*+1/2, −*z*+1/2; (iv) −*x*+2, *y*−1/2, −*z*+3/2.
